# Preservation of kidney function in kidney transplant recipients by alkali therapy (Preserve-Transplant Study): rationale and study protocol

**DOI:** 10.1186/s12882-018-0956-8

**Published:** 2018-07-13

**Authors:** Anna Wiegand, Alexander Ritter, Nicole Graf, Spyridon Arampatzis, Daniel Sidler, Karine Hadaya, Thomas F. Müller, Carsten A. Wagner, Rudolf P. Wüthrich, Nilufar Mohebbi

**Affiliations:** 1 Division of Nephrology, University Hospital Zurich, Zurich, Switzerland; 2 Graf Biostatistics, Wintherthur, Switzerland; 3 Department of Nephrology and Hypertension, University Hospital Berne, Berne, Switzerland; 4 Division of Nephrology, University Hospital Geneva, Geneva, Switzerland; 5 Institute of Physiology, University of Zurich, Zurich, Switzerland

**Keywords:** Metabolic acidosis, Sodium bicarbonate, eGFR, Graft outcome, Graft function

## Abstract

**Background:**

Graft survival after kidney transplantation has significantly improved within the last decades but there is a substantial number of patients with declining transplant function and graft loss. Over the past years several studies have shown that metabolic acidosis plays an important role in the progression of Chronic Kidney Disease (CKD) and that alkalinizing therapies significantly delayed progression of CKD. Importantly, metabolic acidosis is highly prevalent in renal transplant patients and a recent retrospective study has shown that metabolic acidosis is associated with increased risk of graft loss and patient death in kidney transplant recipients. However, no prospective trial has been initiated yet to test the role of alkali treatment on renal allograft function.

**Methods:**

The Preserve-Transplant Study is an investigator-initiated, prospective, patient-blinded, multi-center, randomized, controlled phase-IV trial with two parallel-groups comparing sodium bicarbonate to placebo. The primary objective is to test if alkali treatment will preserve kidney graft function and diminish the progression of CKD in renal transplant patients by assesing the change in eGFR over 2 years from baseline. Additionally we want to investigate the underlying pathomechanisms of nephrotoxicity of metabolic acidosis.

**Discussion:**

This study has the potential to provide evidence that alkali treatment may slow or reduce the progression towards graft failure and significantly decrease the rate of end stage renal disease (ESRD), thus prolonging long-term graft survival. The implementation of alkali therapy into the drug regimen of kidney transplant recipients would have a favorable risk-benefit ratio since alkali supplements are routinely used in CKD patients and represent a well-tolerated, safe and cost-effective treatment.

**Trial registration:**

ClinicalTrials.gov NCT03102996. Trial registration was completed on April 6, 2017.

## Background

The treatment of end-stage chronic kidney disease includes hemodialysis, peritoneal dialysis and kidney transplantation (KTx), while today KTx is the treatment of choice due to better survival, quality of life and cost effectiveness in comparison to maintenance dialysis [1, 2]. Short- and long-term graft survival after KTx have significantly improved within the last decades but there is still a substantial number of patients with declining transplant function and graft loss in the long term. Currently, the mean 20-year graft survival is only 30–40% [3]. Consequently, there is a clear need to develop and test for new therapies that may help to preserve long-term graft function. In addition, identification of reliable candidate biomarkers would allow predicting long-term allograft function or select specific transplant patients that may benefit from new strategies.

Metabolic acidosis (MA) is a well-known complication of CKD and associated with a variety of complications such as uremic bone disease, protein-energy wasting, chronic inflammation, insulin resistance, impairment of myocardial function and disturbances in mineral metabolism, growth hormone and thyroid gland function [4–6]. In addition, observational studies have shown that MA is associated with increased mortality in both, dialysis patients and patients with non-dialysis dependent CKD [7]. Importantly, MA is highly prevalent in renal transplant patients with prevalences reported ranging from 12 to 58% and seems to be consistently more frequent and more severe when compared to non-transplant CKD cohorts with similar degrees of renal function [8–10]. In our clinic, a cross-sectional analysis among 823 unselected kidney transplant patients revealed metabolic acidosis in 58.1% of the patients defined by a serum bicarbonate of < 24 mmol/l indicating a high prevalence of MA in this patient cohort [8]. Notably, eGFR correlated best with serum bicarbonate levels at time of examination. These data could be confirmed by other authors [9] and similar to CKD patients creatinine clearance was markedly lower in the group of transplant patients with MA compared to the non-acidotic patients. Over the past few years several studies have shown that MA plays an important role in the progression of CKD and that low serum bicarbonate levels are associated with a higher risk of progressing to end-stage renal disease [5, 7, 11–15]. Amelioration of MA in CKD patients with alkalinizing therapies delayed progression of GFR loss [16–18]. Furthermore, a recent study has shown for the first time that MA after KTx is associated with increased risk of graft loss and patient death as mentioned before for CKD patients. In this multicenter retrospective cohort study of 2318 patients, a low TCO_2_ level (< 22 mmol/L) 3 months after transplantation was associated with increased risk of graft loss and death-censored graft failure even after adjusting for eGFR [19]. However, to the best of our knowledge, no prospective trial has been initiated yet to test the role of alkali treatment in the prevention of progressive loss of renal allograft function.

Apart from the crucial question if alkali treatment may have a beneficial effect on graft function there are at least two other important aspects of metabolic acidosis after kidney transplantation that are not fully understood yet, namely first the underlying pathomechanisms why MA occurs in KTx patients and second how metabolic acidosis may promote disease progression. It has been reported that MA is more common in patients who received a cadaveric organ compared to a transplant from a living donor [20] but as of today, the influence of donor age, donor renal function and further pre-transplant factors on the occurrence of MA after KTx is still unclear [10]. Other factors such as the intake of calcineurin inhibitors have been shown to be associated with the development of MA by specific changes in tubular epithelial cells which play a role in acid-base regulation [8, 20–24]. In CKD, Wesson et al. investigated intensively the pathogenic effects of endothelin, aldosterone and angiotensin-II on the kidney and its impact on GFR [13, 14, 17, 25–28]. Likewise, a role of the complement system and accumulation of interstitial ammonium had been early proposed to contribute to progression of CKD [29]. Thus, it is essential to investigate the role and in particular the pathomechanisms of MA on graft function and outcome. These findings may help to develop new potential therapeutic targets, such as alkali therapy, that may help to preserve long-term graft survival in this population.

To date, more than 3000 patients with a functioning kidney transplant are living in Switzerland and over 200,000 in the United States [30]. Many of these patients already suffer from decreased graft function which favours the development of MA. Given the expanding pool of CKD patients - including former kidney transplant recipients - an alkali treatment study in kidney transplant patients is of prime importance and has the potential to show that such a treatment may slow or reduce the progression towards graft failure and significantly decrease the rate of end stage renal disease, and therefore prolonging long-term graft survival in KTRs.

## Methods

### Trial design

**Fig. 1:**
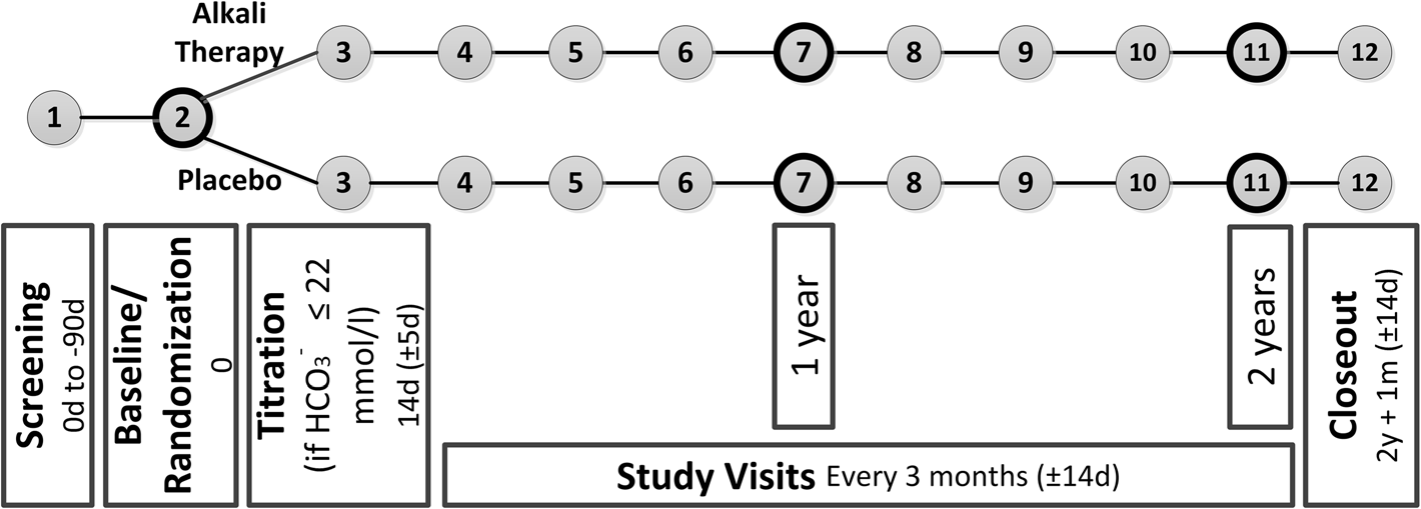
Study Flowchart of the Preserve-Transplant Study. Numbers in circles define each study visit. Study visits 2, 7, and 11 include in addition to blood and spot urine tests 24 h-ambulatory blood pressure monitoring and 24 h urine collection

The Preserve-Transplant Study (PTS) is an investigator-initiated, prospective, patient-blinded, multi-center, randomized, controlled phase-IV trial with two parallel-groups comparing sodium bicarbonate to placebo. The study encompasses 300 patients, ≥ 18 years of age, at least 12 months after renal transplantation and with a serum bicarbonate level ≤ 22 mmol/l. A placebo was chosen as a comparator to the intervention because no equivalent active comparator is existing to date that would qualify as an appropriate comparator. Participants will be randomly allocated to receive sodium bicarbonate or placebo in a 1:1 distribution and will be followed up for 2 years (see Fig. 1). The trial is performed at three transplant centers in Switzerland, in Zurich, Berne and Geneva.

### Objectives and hypothesis

The primary objective of PTS is to test if alkali treatment will preserve kidney graft function and diminish the progression of chronic kidney disease in renal transplant patients by assesing the change in eGFR over 2 years from baseline compared to placebo.

Additionally we want to investigate underlying pathomechanisms of metabolic acidosis and how metabolic acidosis may promote disease progression. A further objective is to identify reliable candidate biomarkers that would allow selecting specific transplant patients that may benefit from alkali therapy.

This study assesses long-term safety of the study drug with a particular focus on new onset and worsening of hypertension, volume overload, and gastrointestinal side effects. Selected adverse events (AEs) and all serious adverse events (SAEs) will be documented regularly and compared between the treatment and placebo group.

### Trial outcomes

The primary outcome of this study is the change in renal function by assessing eGFR within 2 years. eGFR will be determined based on the CKD-EPI creatinine equation. By now, eGFR is the best parameter to assess kidney function and to predict graft and patient survival in kidney transplant recipients [1]. Recent recommendations from the NKF-FDA Scientific Workshop and other publications show that eGFR decline over 2–3 years is an acceptable endpoint for CKD progression trials and clinical transplant research [1, 31–33]. Additionally, several recent studies have used eGFR successfully to investigate the effect of alkali treatment on progression of chronic kidney disease [13, 14, 28]. Moreover, creatinine is a well-established, standardized, and reliable parameter ubiquitously measured in routine clinical practice and compared to inulin or radiolabeled isotopes such as ^125^I Iothalamate or ^51^Cr EDTA very cost-effective and easy available.

Important secondary outcomes include changes in serum bicarbonate levels, pH, sodium, potassium, and proteinuria measured as protein/creatinine or albumin/creatinine ratio in spot urine. As a safety outcome, 24-h ambulatory blood pressure monitoring will be performed at the beginning of the study and after 1 and 2 years to evaluate the effect of sodium bicarbonate on blood pressure. All outcomes of interest are shown in Table 1**.**

**Table 1:** Study Outcomes

Primary Outcome	Change in renal function by assessing eGFR (CKD-EPI) within 2 years
Important Secondary Outcomes	Changes in serum bicarbonate levels, pH, sodium, potassium, and proteinuria measured as protein/creatinine or albumin/creatinine ratio in spot urine
Safety Outcome	Twenty-four-hour ambulatory blood pressure monitoring will be performed at the beginning of the study and after 1 and 2 years
Other Outcomes of Interest	• Changes in measurement of specific acid base transport proteins by urinary exosome collection • Changes in urinary ammonium excretion, inflammatory markers such as complement factors, and hormones involved in tubulo-interstitial nephritis/fibrosis such as endothelin and aldosterone • Histopathological grade of tubulo-interstitial fibrosis according to BANFF classification in kidney biopsies (where available) will be analyzed in both arms • Systemic calcification propensity in blood will be determined by the *T*_*50*_-Test (A test measuring the transformation time (*T*_*50*_) of primary to secondary calciprotein particles, where a long delay of *T*_*50*_ indicates a high residual capacity of the patients’ serum to prevent the formation of secondary calciprotein particles and is therefore a sign of an intact endogenous defense against calcification) [34] • Change of bone density and rejection rate

### Study population

Kidney transplant patients will be recruited from all study centers according to the inclusion and exclusion criteria (Table 2).

**Table 2:** Inclusion and Exclusion Criteria of the Preserve-Transplant Study

Inclusion Criteria	
• Informed consent as documented by signature	
• Age ≥ 18 years and able to give informed consent	
• ≥ 12 months after renal transplantation	
• Stable clinical condition	
• Stable graft function over the last 6 months (creatinine changes ±15%)	
• eGFR between 15 and 89 ml/min/1.73 m^2^	
• At least one serum bicarbonate measured ≤22 mmol/l within the last 6 months	
Exclusion Criteria	
• Uncontrolled hypertension or use of > 4 antihypertensive agents	
• Uncontrolled heart failure	
• Serum potassium < 3.0 mmol/l	
• Serum sodium > 150 mmol/l	
• Use of alkali in the preceding 4 weeks	
• Use of mineralocorticoid antagonists, topiramate, carbo anhydrase inhibitors or any drugs with similar effects	
• History of noncompliance with clinic visits	
• Hereditary fructose intolerance	
• Known hypersensitivity or allergy to the drug used in this study or to peanut, sorbitol, and soy	
• Pregnancy or breastfeeding	
• Intention to become pregnant during the course of the study	
• Lack of safe contraception, defined as: Female participants of childbearing potential, not using and not willing to continue using a medically reliable method of contraception for the entire study duration, such as oral, injectable, or implantable contraceptives, or intrauterine contraceptive devices, or who are not using any other method considered sufficiently reliable by the investigator in individual cases. Please note that female participants who are surgically sterilised / hysterectomised or post-menopausal for longer than 2 years are not considered as being of child bearing potential.	
• Suspected drug or alcohol abuse	
• Inability to follow the procedures of the study, e.g. due to language problems, psychological disorders, dementia, etc. of the participant	
• Enrolment of the investigator, his/her family members, employees and other dependent persons	

### Study enrolment, randomization and blinding

Potential candidates will be identified from the electronic hospital databases. After signature of the informed consent and successful screening patients will be randomized in a 1:1 ratio stratified by each study center and gender using a permuted block design. The block size will be concealed until the primary endpoint will be analyzed. The randomization lists remain within the electronic data management tool for the whole duration of the study. Thus, randomization will be conducted without any influence of the principal investigators or other study personnel. The study is single-blind on the patient side.

### Biobank

Biobank samples are obtained at every study visit in all study centers and stored at − 80 °C for 5 years after termination of the study according to Swiss legal requirements. All samples or genetic data will be stored and used for future research purposes with the participant’s consent.

### Investigational medicinal product

Nephrotrans® (sodium hydrogen carbonate, ATC-Code: A02AH) has been approved and used since 1986 for the treatment of metabolic acidosis. Nephrotrans® is a safe, well-tolerated drug with very few and well-studied side effects. The study drug will be provided by SALMON Pharma GmbH in its commercially available formulation, used orally in this study and will be given at an initial dose of 500–1000 mg sodium hydrogen carbonate thrice daily. The dosage is chosen according to the body weight of the patient at baseline visit (< 70 kg or ≥ 70 kg). If serum bicarbonate remains ≤22 mmol/l the dosage of sodium hydrogen bicarbonate will be titrated once to 1000–1500 mg thrice daily as for placebo too at visit 3 after 2 weeks. All patients will be blinded to the identity of the study drug. The study product will be administered for a total of 2 years. The placebo is also provided by SALMON Pharma GmbH and used as a comparator to the intervention in this study. All patients will have the best standard care for kidney transplant patients.

### Informed consent

Written informed consent for participation will be obtained from all participants before any study related actions are taken. Patients will be informed that withdrawal from the study is possible at any time without giving reasons.

### Approvals

The study has been approved by the Cantonal Ethics Committee of Zürich, Berne and Geneva and Swissmedic as the competent authority. The study has been registered on ClinicalTrials.gov (NCT03102996). The project will be conducted in line with the Declaration of Helsinki as well as all national legal and regulatory requirements. In addition all researchers will follow the GCP guidelines. All non-substantial and substantial amendments of the protocol will be reported to the ethics committees and the competent authorities in Switzerland.

### Criteria for discontinuation

Patients will be withdrawn from participation in the trial if they withdraw their consent or the investigator or sponsor withdraws the patient because of malcompliance with the study intervention. Additionally, if serum bicarbonate exceeds 35 mmol/l or falls below 16 mmol/l the patient will have to be excluded from the study for safety reasons. Pregnancy in women or a significant violation of the protocol as judged by the coordinating investigator also will lead to withdrawal of the participant. Patients will be withdrawn from study treatment permanently if a severe allergic or hypersensitivity reaction to the drug occurs or if the investigator feels that the treatment is harmful to the subject. In case of a treatment-related SAE the study drug will also be withdrawn from the patient. A study patient who discontinues study participation prematurely for any reason is defined as dropout if the subject has already been randomized. A study patient who terminates the study before randomization is regarded as a screening failure.

### Monitoring

Quality assurance will be based on a central and on-site monitoring. The central monitoring encompasses monitoring of data entry progress. Trial sites will also be regularly visited by independent monitors. Visits will focus on controlling regulatory compliance, monitoring of processes, and verification of data that cannot be monitored centrally.

### Data management

In the Preserve-Transplant study, all data will be entered electronically using an internet-based secure data base (secuTrial®) developed in agreement with the Good Clinical Practice (GCP) guidelines. Data integrity is enforced through a variety of mechanisms. Regular backup of the data is assured.

### Statistical analysis

The analysis population consists of all patients who were randomized and have valid data for baseline and at least one follow-up. The primary and secondary endpoints will be analyzed with linear mixed models. Moreover, exploratory subgroup analyses are planned with respect to baseline eGFR to evaluate whether subgroups with lower baseline eGFR will show a better response to alkali treatment compared to subgroups with higher baseline eGFR. Any further details will be specified in the statistical analysis plan.

### Sample size

Based on a retrospective analysis of our own cohort, it was assumed that the mean annual eGFR decline in the placebo group will be 1.5 ml/min/1.73 m^2^ and that this decline can be reduced to 0 ml/min/1.73 m^2^ in the treatment group as a result of alkali therapy based on previously published data for CKD patients with metabolic acidosis. Power was determined on the basis of a two-sided, two-sample t-test of the slopes over 2 years. Group sizes of 150 will achieve 82% power to find a minimum detectable difference in the mean slope over 2 years of − 3.0 with estimated group standard deviations of 9.0 and with a significance level of 0.05 using a two-sided two-sample t-test. Therefore, a total number of 300 patients for the entire study is planned.

## Discussion

Metabolic acidosis is a serious complication in patients with CKD with deleterious effects on kidney function and patient survival. A recent retrospective study has evidenced that MA is also associated with increased risk of graft loss and patient mortality in kidney transplant recipients [19]. Although several studies have demonstrated a beneficial role for alkali therapy on the progression of CKD [5, 7, 11–15], no data is available if alkali treatment may also allow to better preserve renal allograft function in kidney transplant recipients with metabolic acidosis.

The Preserve-Transplant Study is the first investigator-initiated prospective clinical trial that aims to test whether alkali treatment will help preserving kidney graft function and diminish the progression of chronic kidney disease in renal transplant patients. Thereby, alkali therapy may have the potential to become the first evidence-based adjuvant therapy for the preservation of graft function in kidney transplant patients.

Consequently, the results of the PTS may provide evidence to adapt current recommendations and guidelines for the routine treatment of KTRs suffering from metabolic acidosis. The increasing demand for donor organs worldwide calls for solutions to help to prolong graft survival. The implementation of alkali therapy into the drug regimen of kidney transplant recipients would have a favorable risk-benefit ratio since alkali supplements are routinely used in CKD patients and represent a well-tolerated, safe and cost-effective treatment.

## Trial status and dissemination

The Preserve-Transplant randomized controlled clinical study has received governance approval and is registered at ClinicalTrials.gov (NCT03102996). The trial started recruitment in June, 2017. The study will end in June 2021. First results from this trial are expected in the first quarter of 2022. Publications will be submitted to peer-reviewed journals and the results will be presented at national and international scientific conferences.

## Abbreviations

AE: Adverse event
CKD: Chronic kidney disease
eGFR: Estimated Glomerular Filtration Rate
ESRD: End stage renal disease
GCP: Good clinical practice
KTRs: Kidney transplant recipients
KTx: Kidney transplantation
MA: Metabolic acidosis
PTS: Preserve-Transplant Study
SAE: Serious adverse event
